# Conjugation of wildtype and hypoallergenic mugwort allergen Art v 1 to flagellin induces IL-10-DC and suppresses allergen-specific TH2-responses *in vivo*

**DOI:** 10.1038/s41598-017-11972-w

**Published:** 2017-09-18

**Authors:** Stefan Schülke, Kirsten Kuttich, Sonja Wolfheimer, Nadine Duschek, Andrea Wangorsch, Andreas Reuter, Peter Briza, Isabel Pablos, Gabriele Gadermaier, Fatima Ferreira, Stefan Vieths, Masako Toda, Stephan Scheurer

**Affiliations:** 1 Section Molecular Allergology, Paul-Ehrlich-Institut, Langen, Hessen Germany; 2 Division of Allergology, Paul-Ehrlich-Institut, Langen, Hessen Germany; 3 Department of Molecular Biology, Division of Allergy and Immunology, University of Salzburg, Salzburg, Austria

## Abstract

Allergies to weed pollen including members of the *Compositae* family, such as mugwort, ragweed, and feverfew are spreading worldwide. To efficiently treat these newly arising allergies, allergen specific immunotherapy needs to be improved. Therefore, we generated novel vaccine candidates consisting of the TLR5-ligand Flagellin A from *Listeria* and the major mugwort allergen Art v 1 including either the wild type Art v 1 sequence (rFlaA:Artv1) or a hypoallergenic variant (rFlaA:Artv1^hyp^) with reduced IgE-binding capacity. Immune modulating capacity of these constructs and respective controls was evaluated *in vitro* and *in vivo*. Incorporation of hypoallergenic Art v 1 derivative did not interfere with the resulting fusion proteins’ immune stimulatory capacity. Both rFlaA:Artv1 and rFlaA:Artv1^hyp^ induced a prominent, mTOR-dependent, IL-10 secretion from murine dendritic cells, and suppressed allergen-specific TH2-cytokine secretion *in vitro* and *in vivo*. Both conjugates retained the capacity to induce rFlaA-specific antibody responses while efficiently inducing production of Art v 1-specific IgG1 and IgG2a antibodies in mice. Interestingly, only the suppression of TH2-cytokine secretion by rFlaA:Artv1 (but not rFlaA:Artv1^hyp^) was paralleled by a strong secretion of IFN-γ. In summary, we provided evidence that incorporating hypoallergens into flagellin:allergen fusion proteins is a suitable strategy to further improve these promising vaccine candidates.

## Introduction

The prevalence of IgE-mediated allergies has increased worldwide, probably caused by factors such as changes in lifestyle, epigenetics, and spreading of allergens due to global climate changes. According to the World Allergy Organization (WAO) White Book on Allergy 2011–2012, about 30–40% of the world’s population is affected by one or more allergic conditions^1^. Here, both the severity and complexity of allergic diseases particularly in children and young adults have been shown to increase, causing a significant social and economic burden for individuals and societies^1^.

An arising source of weed pollen allergies are members of the *Compositae* family, including mugwort, ragweed, and feverfew^2^. Mugwort is domestic in Northern and Central Europe, the US, and several parts of Asia^3^. Already 10–14% of weed pollen sensitized subjects in Europe are affected by allergies to mugwort pollen^4^. Furthermore, the prevalence of sensitization to mugwort pollen in Germany was found to be 4.5%^5^ and 7.2% for Art v 1 in Austrian adolescents^6^. Moreover, a strongly increased geographical spreading of the genus *Artemisia* (mugwort)^7^ due to climate changes has been observed, which will likely result in further increased rates of sensitization towards *Artemisia* allergens in the future.

The major mugwort allergen Art v 1^8–10^ is an attractive target molecule for the development of genetically engineered allergen vaccines. Art v 1 consists of 108 amino acids divided into a folded defensin-like domain, stabilized by intramolecular cysteine bonds, and a flexible hydroxyproline-rich part^8–10^. IgE-antibodies directed against Art v 1 predominantly recognize the defensin-like domain, which also harbors the immunodominant human T-cell epitope at amino acid position 25 to 36^10–12^.

While the introduction of vaccines has strongly improved our treatment options for many infectious diseases, the search for efficacious vaccines to treat allergic diseases remains a major task. Currently, the only established therapy option for the treatment of allergies is conventional allergen immunotherapy (AIT) with allergen extracts. However, AIT is not convenient for patients due to a multi-year treatment regimen, for some allergies only partially efficacious, and can be hampered by severe allergic side effects^13,14^. To improve AIT, novel vaccine candidates and accompanying adjuvants that increase efficacy while decreasing unwanted adverse-effects are needed^15^. Strategies currently investigated for improved treatment of allergic diseases include for example the usage of different adjuvants, more thoroughly defined recombinant allergens or hypoallergenic allergen-derivatives and allergen-derived peptides combining preserved T cell reactivity with the reduced capacity to activate B cells^16^.

Among the different adjuvants currently tested TLR-ligands are promising candidates to modulate allergen-specific TH2 responses because of their intrinsic ability to induce robust, mostly TH1-biased, immune responses. In this context, the TLR5-ligand flagellin is of special interest, since its proteinous nature enables the generation of fusion proteins consisting of flagellin and antigen using recombinant DNA technologies. Flagellin is a well-known bacterial motility protein with an established adjuvant potential^17–19^. In theory, fusion proteins combining antigen and flagellin (as adjuvant) into a single molecule enable efficient targeting of antigens to TLR5 positive APCs as well as the processing and presentation of the fused antigen in the context of the flagellin-mediated APC activation^20^. Indeed, a plethora of studies suggest that flagellin-containing fusion proteins efficiently induce protective humoral and cell mediated immune responses against the conjugated (often otherwise poorly immunogenic) antigen and therefore have potential as vaccine candidates for many different types of infectious diseases^21–28^ and allergies^20,29^. In previous studies, we have shown that application of a fusion protein consisting of flagellin A (FlaA) derived from *Listeria* and the model allergen ovalbumin resulted in the generation of IL-10 producing tolerogenic dendritic cells and was able to prevent the establishment of intestinal allergy in an experimental mouse model^20,29^.

Therefore, the aim of this study was to generate novel vaccine candidates consisting of the TLR5 ligand and the major mugwort allergen Art v 1 and investigate their immune modulating capacity both, *in vitro* and *in vivo*. In order to further improve the safety profile of such fusion proteins, we generated two different fusion proteins: rFlaA:Artv1 incorporating the wild type Art v 1 sequence and rFlaA:Artv1^hyp^ incorporating a hypoallergenic Art v 1 sequence in which seven out of eight cysteine residues were exchanged to serine residues in order to disrupt conformational IgE epitopes^30^. Of note, upon incorporation of the hypoallergenic Art v 1 sequence, the resulting fusion protein retained its capacity to activate, both murine and human dendritic cells and to suppress Art v 1-induced TH2 cytokine secretion *ex vivo* and *in vivo*. Therefore, we think that incorporating hypoallergens into flagellin:allergen fusion proteins is a viable strategy to further improve the safety profile of these promising vaccine candidates.

## Results

### rFlaA, rFlaA:Artv1, and rFlaA:Artv1^hyp^ were generated with high purity and biological activity

**Figure 1:**
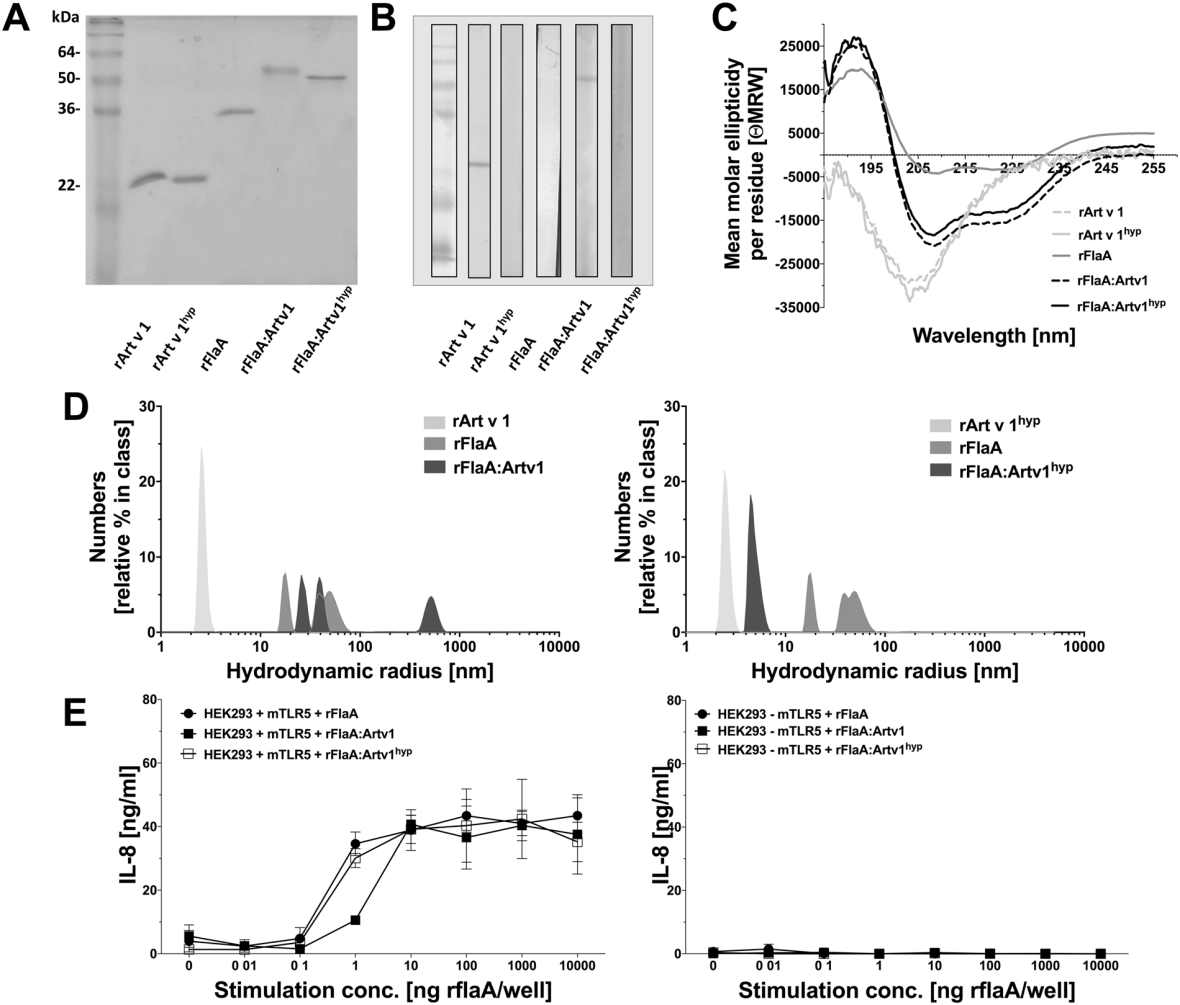
rFlaA, rFlaA:Artv1, and rFlaA:Artv1^hyp^ were generated with high purity and biological activity. Cropped coomassie staining (**A**) raw data available in Repos Fig. S5), immunoblot (**B**) and circular dichroism (CD) spectroscopy (**C**) of final protein preparations. Hydrodynamic radii of the different proteins were determined by dynamic light scattering analysis (**D**) and potency of the rFlaA-containing constructs to activate TLR5 was evaluated by determination of IL-8 secretion from murine TLR5-transfected HEK293 cells stimulated for 22 h with the different constructs (**E**). Data are mean values of ten measurements (**D**) or mean values ± SD of three (**E**) independent experiments ± SD.

Using the described methods for protein expression and purification all recombinant proteins used in this study were generated with high purity and displayed the expected apparent molecular mass (rArt v 1: 24 kDa, rArt v 1^hyp^: 24 kDa, rFlaA: 30.6 kDa, rFlaA:Artv1: 52 kDa, and rFlaA:Artv1^hyp^: 52 kDa, Fig. 1A). Identity of the fusion proteins was confirmed by tandem mass spectrometry after tryptic digests (data not shown). IgE-immunoblot analysis using sera of mugwort pollen-allergic individuals showed that IgE-reactivity with Art v 1 was retained for rFlaA:Artv1, whereas no IgE reactivity was observed for rFlaA and the hypoallergenic derivatives rArt v 1^hyp^ and rFlaA:Artv1^hyp^ (Fig. 1B). Analysis of secondary structure elements by circular dichroism (CD) spectroscopy revealed both ω-shaped curve progressions and minima typical for mainly α-helical proteins for rFlaA, rFlaA:Artv1, and rFlaA:Artv1^hyp^ whereas rArt v 1 and rArt v 1^hyp^ displayed u-shaped curves typical of random coil structures (Fig. 1C). The circular dichroism spectra obtained for rArt v 1 and rArt v 1^hyp^ are in accordance with previous reports describing dominating random coil structures of rArt v 1 likely caused by its high proline content^3,30^. For further protein characterization, hydrodynamic radii (R_H_) of the different proteins were assessed by dynamic light scattering analysis (Fig. 1D). While for rArt v 1 (R_H_ = 2.44 nm), rArt v 1^hyp^ (R_H_ = 2.56 nm), and rFlaA:Artv1^hyp^ (R_H_ = 4.46 nm) single radii were observed, for rFlaA (R_H_ = 17.82 + 39.08 + 49.24 nm), and rFlaA:Artv1 (R_H_ = 25.78 + 39.08 + 519.6 nm) multiple fractions with considerably higher hydrodynamic radii were detected, suggesting the formation of high molecular aggregates.

Capacity to the different proteins to activate the target receptor TLR5 was determined using HEK293 reporter cells stably transfected with murine TLR5 (Fig. 1E). Here, both rFlaA-containing constructs dose-dependently induced comparable levels of IL-8 secretion and no significant differences in the capacity of rFlaA, rFlaA:Artv1, or rFlaA:Artv1^hyp^ to activate TLR5 were detectable (Fig. 1E). However, at a stimulation concentration equimolar to 1 µg/ml of rFlaA, rFlaA:Artv1 consistently induced lower IL-8 responses in comparison to those observed for rFlaA or rFlaA:Artv1^hyp^ (Fig. 1E). As expected, none of the tested proteins resulted in IL-8 secretion from HEK293 cells lacking TLR5-expression (Fig. 1E). In summary, all proteins used in this study were generated with high purity and retained their ability to activate the target receptor TLR5.

### rFlaA:Artv1 and rFlaA:Artv1^hyp^ potently activate mDC leading to both pro- and anti-inflammatory cytokine secretion

**Figure 2:**
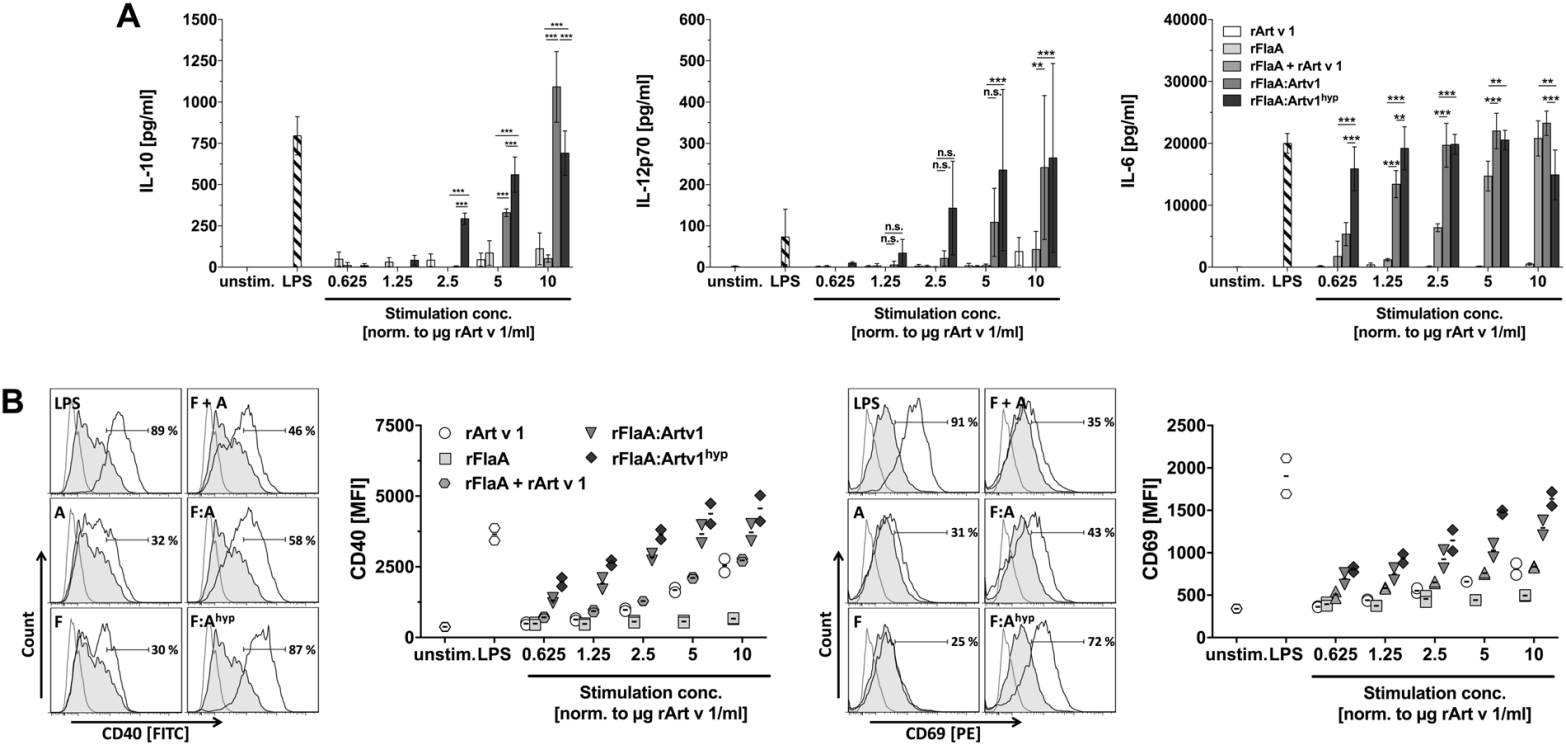
rFlaA:Artv1 and rFlaA:Artv1^hyp^ potently activate mDC leading to both pro- and anti-inflammatory cytokine secretion. BALB/c-bone marrow-derived mDCs were stimulated *in vitro* with 10 µg/ml LPS or equimolar amounts of rArt v 1, rFlaA, rFlaA + rArt v 1, rFlaA:Artv1, or rFlaA:Artv1^hyp^ for 24 h and cytokine levels in culture supernatants were quantified by ELISA (**A**). BALB/c mDCs were stimulated for 24 h as described in (**A**), gated on CD11b^+^CD11c^+^B220^−^ mDCs, and analyzed for CD40 and CD69 expression by flow cytometry (**B**). Representative histograms of CD40 and CD69 expression on mDCs stimulated with protein amounts equimolar to 2.5 µg Art v 1/ml with frequencies of CD40^high^/CD69^high^ activated CC11c^+^CD11b^+^B220^−^ mDCs and MFI values for CD40/CD69 expression are depicted in (**B**). Data are mean values ± SD of two (**B**) or three (**A**) independent experiments.

To investigate the immune modulating capacity of the fusion proteins, bone marrow-derived murine myeloid dendritic cells (mDC) were stimulated with equimolar amounts of the different proteins and analyzed for cytokine secretion and mDC activation (Fig. 2). Stimulation with rFlaA:Artv1 and rFlaA:Artv1^hyp^ induced a dose-dependent and significantly higher secretion of pro- (IL-12p70) and anti-inflammatory (IL-10) cytokines from BALB/c-derived mDCs, which was not observed for equimolar amounts of both proteins alone or provided as a non-fused mixture (Fig. 2A). Here, higher concentrations (>concentrations equimolar to 2.5 µg rArt v 1/ml) of the mixture of rFlaA + Artv1 were required to induce a substantial IL-6 secretion (Fig. 2A). Thresholds for the induction of mDC-derived cytokine secretion were different for IL-6 (rFlaA + Artv1: 2.5 µg/ml, rFlaA:Artv1: 0.625 µg/ml, rFlaA:Artv1^hyp^: 0.625 µg/ml), IL-10 (rFlaA:Artv1: 5 µg/ml, rFlaA:Artv1^hyp^: 2.5 µg/ml), and IL-12p70 (rFlaA:Artv1: 5 µg/ml, rFlaA:Artv1^hyp^: 2.5 µg/ml, Fig. 2A). Similar results were obtained for TNF-α secretion (data not shown). In accordance with the results obtained from BALB/c bone marrow-derived mDC both fusion proteins induced a higher secretion of pro- (IL-6 and IL-12p70) and anti-inflammatory (IL-10) cytokines from C57Bl/6 bone marrow-derived mDCs than the respective controls (Supplementary Fig. S1). Moreover, the overall levels of the induced cytokines were comparable between C57Bl/6 and BALB/c-derived mDC (Supplementary Fig. S1).

In line with the pronounced secretion of pro- and anti-inflammatory cytokines, a strong upregulation of the maturation and costimulatory molecules CD40 and CD69 was detected on CD11b^+^CD11c^+^B220^−^ mDCs (BALB/c) stimulated with rFlaA:Artv1 or rFlaA:Artv1^hyp^ (and to a lesser extend the equimolar mixture of both proteins rFlaA + Artv1) (Fig. 2B). Determination of mDC viability upon stimulation with the different proteins showed less than 2% dead cells in both unstimulated cells and cells stimulated with the different proteins (Supplementary Fig. S2), indicating that in the concentrations used none of the tested proteins had a toxic effect on the tested mDC.

In summary, compared to the mixture of non-fused components, stimulation with both fusion proteins strongly promoted mDC activation and led to increased production of pro- and anti-inflammatory cytokines.

### rFlaA:Artv1 and rFlaA:Artv1^hyp^ suppress TH2-cytokine production in a co-culture of mDC and Art v 1-specific TH2-biased T cells

**Figure 3:**
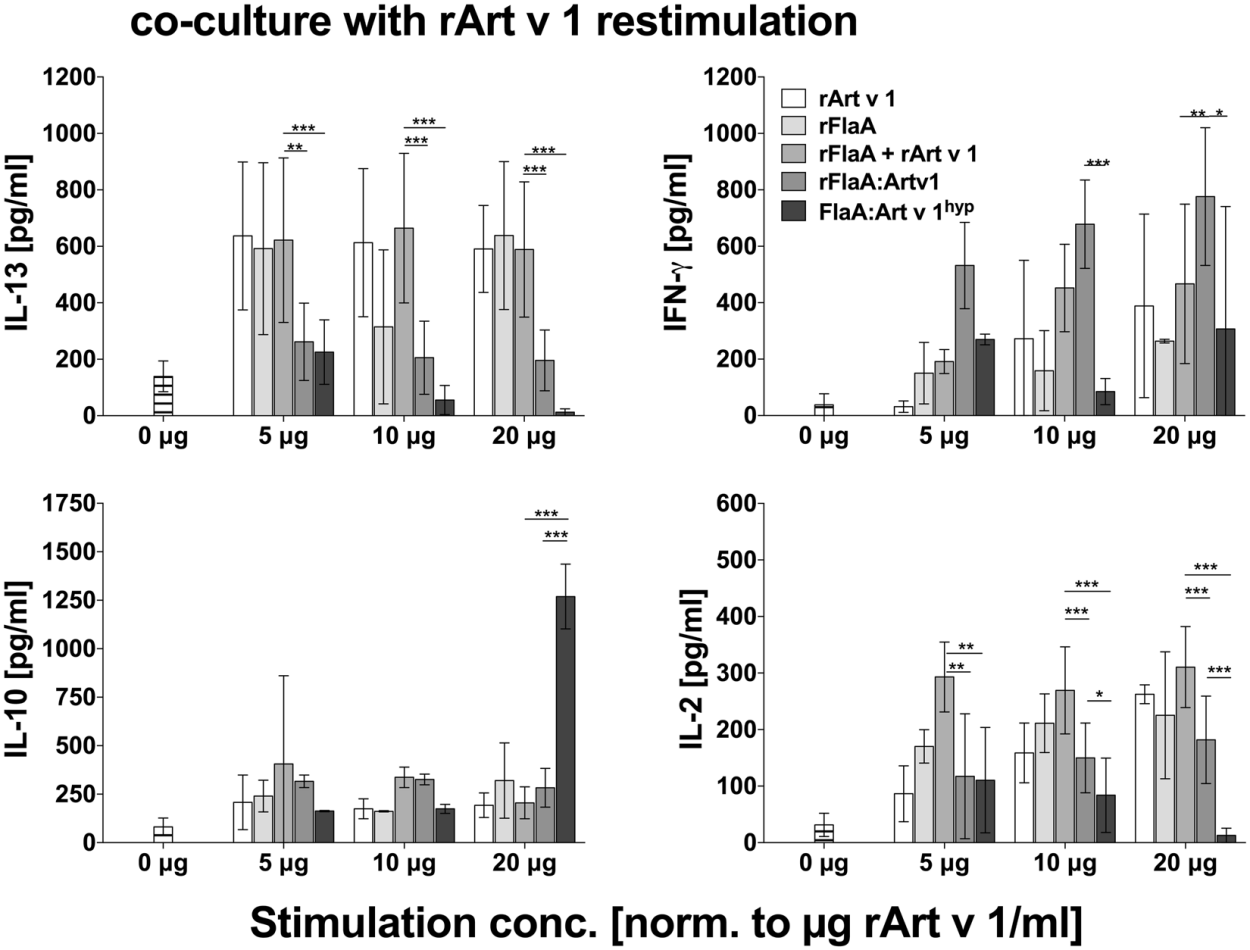
Both fusion proteins suppress TH2-cytokine production in a co-culture of mDC and Art v 1-specific, TH2-biased T cells. BALB/c bone marrow-derived mDCs were co-cultured with *ex vivo*-isolated CD4^+^ T cells from Art v 1-immunized BALB/c mice and stimulated with the indicated proteins in the presence of rArt v 1- to induce Ag-specific recall responses. Levels of IL-2 in culture supernatants were determined 24 h post-stimulation, IFN-γ, IL-13, and IL-10 levels 72 h post-stimulation by ELISA. Data are mean values ± SD of three independent experiments.

Next we investigated the impact of rFlaA:Artv1- and rFlaA:Artv1^hyp^-mediated activation of mDCs on the induction of adaptive immune responses and their ability to modulate allergen-specific T cell responses. We performed co-culture experiments using mDCs in combination with *ex vivo* isolated, TH2-biased CD4^+^ T cells from rArt v 1 + Alum sensitized animals and checked whether both fusion proteins were able to modulate TH2-responses induced by Art v 1-re-stimulation in these co-cultures (Fig. 3). Whereas IL-13 (a typical marker cytokine of respiratory allergy) was induced upon re-stimulation of mDC:TC co-cultures with rArt v 1, co-administration of rFlaA:Artv1 or rFlaA:Artv1^hyp^, but not with both proteins provided separately or as a mixture, was able to dose dependently and significantly suppress rArt v 1-induced IL-13 secretion (Fig. 3). Interestingly, this suppression of TH2 cytokine secretion was paralleled by high levels of IFN-γ secretion in co-cultures stimulated with rFlaA:Artv1, whereas anti-inflammatory IL-10 secretion was induced by rFlaA:Artv1^hyp^ (20 µg/ml) (Fig. 3). Here, IL-10 secretion was associated with a suppression of IL-2 secretion (a marker of T cell activation and proliferation) (Fig. 3).

Similar results were obtained from DC:TC co-cultures stimulated with the different proteins in the absence of additional re-stimulation with rArt v 1 (Supplementary Fig. S3). Interestingly, rFlaA:Artv1^hyp^-induced IL-10 and IFN-γ secretion were inversely regulated. Of note, stimulation with both Art v 1 fusion proteins resulted in considerably lower levels of IL-2 and IL-13 secretion from the rArt v 1-specific TH2-biased T cells compared to its unfused counterpart (Supplementary Fig. S3). Accordingly performed stimulations of mDCs alone in the absence of CD4^+^ T cells showed, that the cytokines IL-13, IFN-y, and IL-2 detected in the corresponding co-cultures were only produced in mDC:T cell co-cultures whereas the observed IL-10 secretion could be attributed to the mDCs within the mDC:CD4 T cell co-cultures (data not shown).

In summary, both fusion proteins were able to efficiently suppress TH2 cytokine secretion from TH2-biased T cells in the presence of their specific antigen.

### Art v 1-induced IL-13 secretion is suppressed after vaccination with rFlaA:Artv1 and rFlaA:Artv1^hyp^

**Figure 4:**
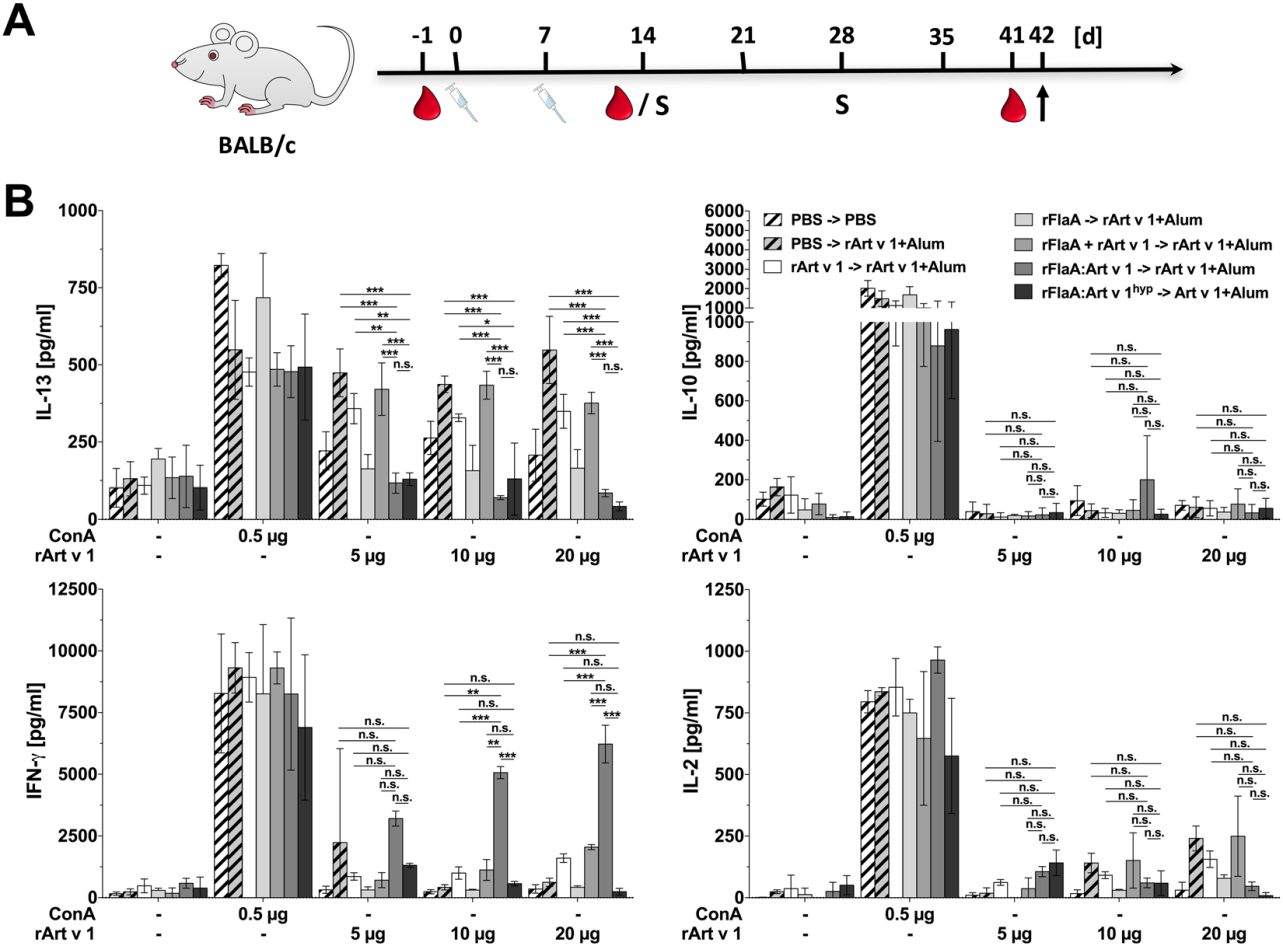
Art v 1-induced IL-13 secretion is suppressed after vaccination with rFlaA:Artv1 and rFlaA:Artv1^hyp^. BALB/c mice were treated two times (i.p.) with the indicated proteins (all equimolar to 10 µg rArt v 1) or PBS, followed by two i.p.-sensitizations with Art v 1+ Alum in a 2-week interval (**A**). CD4^+^ TCs were isolated on day 42 and co-cultured with BALB/c bone-marrow-derived mDCs and re-stimulated *in vitro* with rArt v 1. After 24 (IL-2) and 72 h (all other cytokines), supernatants were collected and analyzed for levels of IL-2 and IL-13, IL-10, and IFN-γ (**B**). Spleens of three animals per group were pooled for the isolation of CD4^+^ T cells. Data are mean results of two independent studies ± SD.

In order to study the immune modulating capacity of both fusion proteins *in vivo*, we investigated if prophylactic vaccination with the different proteins was able to modulate TH2-biased T cell responses induced by subsequent Art v 1 + Alum sensitization (Fig. 4A). CD4^+^ T cells were isolated two weeks after the final sensitization, co-cultured with BALB/c mDCs, and checked for cytokine profile upon rArt v 1 re-stimulation (Fig. 4B).

Prophylactic vaccination with rFlaA:Artv1 and rFlaA:Artv1^hyp^ (and to a lesser extent rFlaA) was able to suppress the rArt v 1-mediated induction of IL-13 and IL-2 secretion from mDC:TC co-cultures (Fig. 4B). Moreover, rArt v 1-induced IL-5 secretion was suppressed after vaccination with both fusion proteins (Supplementary Fig. S4). Interestingly, rArt v 1-induced IL-5 secretion was also suppressed in all other vaccinated groups (Supplementary Fig. S4). Moreover, in this co-culture system generally no IL-4 secretion was detectable, probably due to its low stability in the applied cell culture medium (data not shown). Interestingly, for co-cultures of T cells isolated from animals vaccinated with rFlaA:Artv1 the suppression of cytokine secretion was paralleled by a highly significant increase in IFN-γ levels which was not observed with T cells isolated from animals vaccinated with rFlaA or rFlaA:Artv1^hyp^. Here, vaccination with neither rArt v 1 nor the mixture of rFlaA + rArt v 1 did result in a comparable suppression of IL-13 and IL-2 secretion (Fig. 4B). Moreover, *in vitro* re-stimulation with rArt v 1 alone did not result in high levels of IL-10 secretion (Fig. 4B).

In summary, these results show that prophylactic vaccination with both fusion proteins was able to efficiently suppress the differentiation of TH2 cells *in vivo*.

### Both Art v 1 fusion proteins efficiently induce Art v 1-specific IgG production

**Figure 5:**
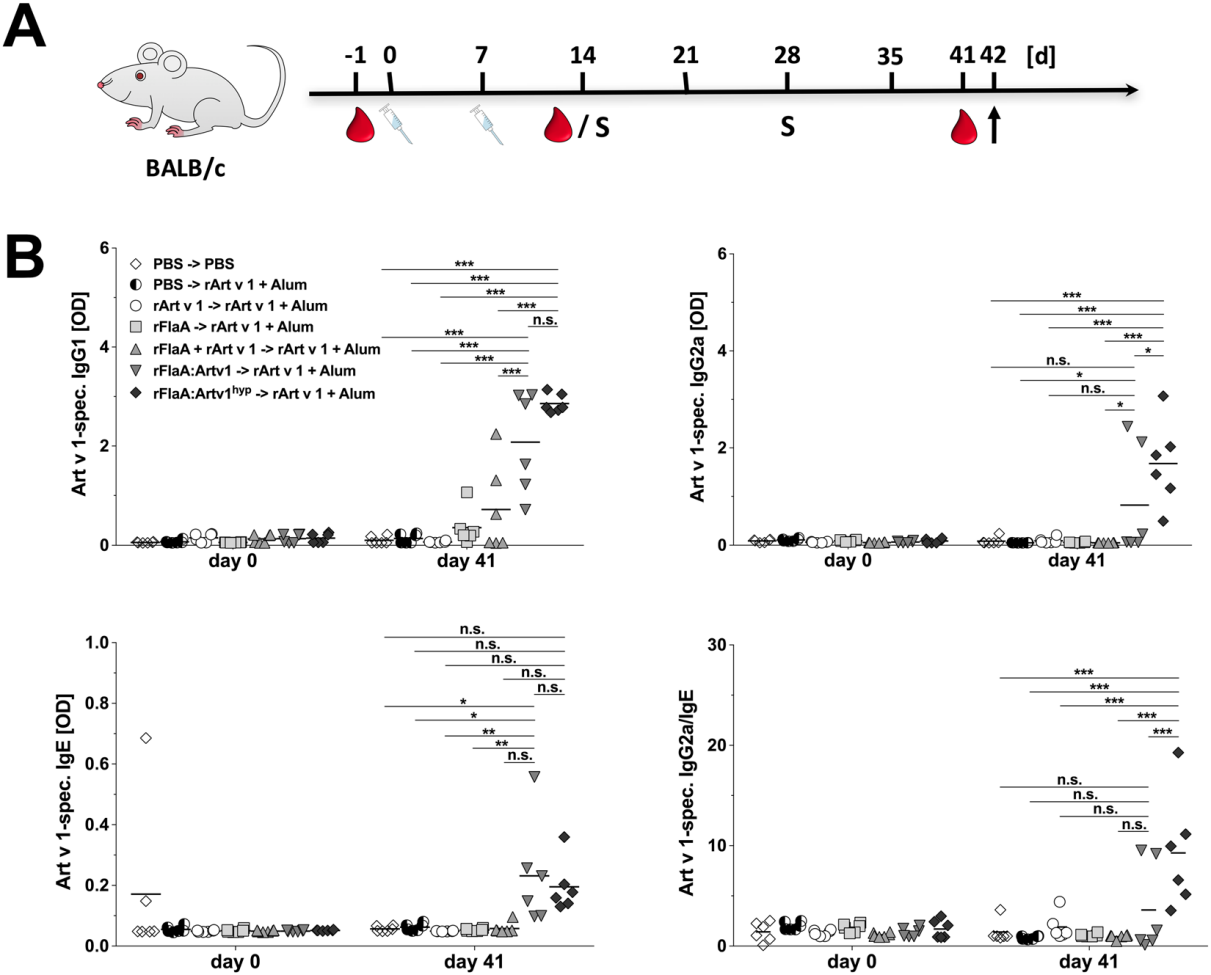
Both Art v 1 fusion proteins induce Art v 1-specific antibody production. BALB/c mice were treated two times (i.p.) with the indicated proteins (all equimolar to 10 µg rArt v 1) or PBS, followed by two i.p.-sensitizations with Art v 1+ Alum (S) in a 2-week interval (**A**). On days 0, 14, and 41 serum samples were collected and analyzed for Art v 1-specific IgG1-, IgG2a-, and IgE-levels by ELISA (**B**). Data are mean results of two independent studies with n = 3 mice/group and study.

Vaccination with both fusion proteins induced significantly increased levels of rArt v 1-specific IgG1 and IgG2a antibodies compared to animals vaccinated with the respective controls (Fig. 5). Here, significantly lower but still detectable levels of rArt v 1-specific IgG1 antibodies were also observed in animals vaccinated with rFlaA + rArt v 1 but not in the other treatment groups (Fig. 5B). rArt v 1-specific IgG2a production was only detectable in animals vaccinated with either rFlaA:Artv1 or rFlaA:Artv1^hyp^ (Fig. 5B). Compared to this prominent induction of IgG antibodies, overall levels of rArt v 1-specific IgE antibodies were very low (OD range: 0.05–0.3, Fig. 5B). Accordingly, Art v 1-specific IgG2a/IgE ratios were only enhanced in animals vaccinated with both fusion proteins (rFlaA:Artv1: two out of six mice, rFlaA:Artv1^hyp^: all six mice, Fig. 5B).

### rFlaA:Artv1 and rFlaA:Artv1^hyp^ induce both rFlaA- and rArt v 1-specific antibody responses while reducing TH2-cytokine secretion upon re-stimulation with rFlaA

**Figure 6:**
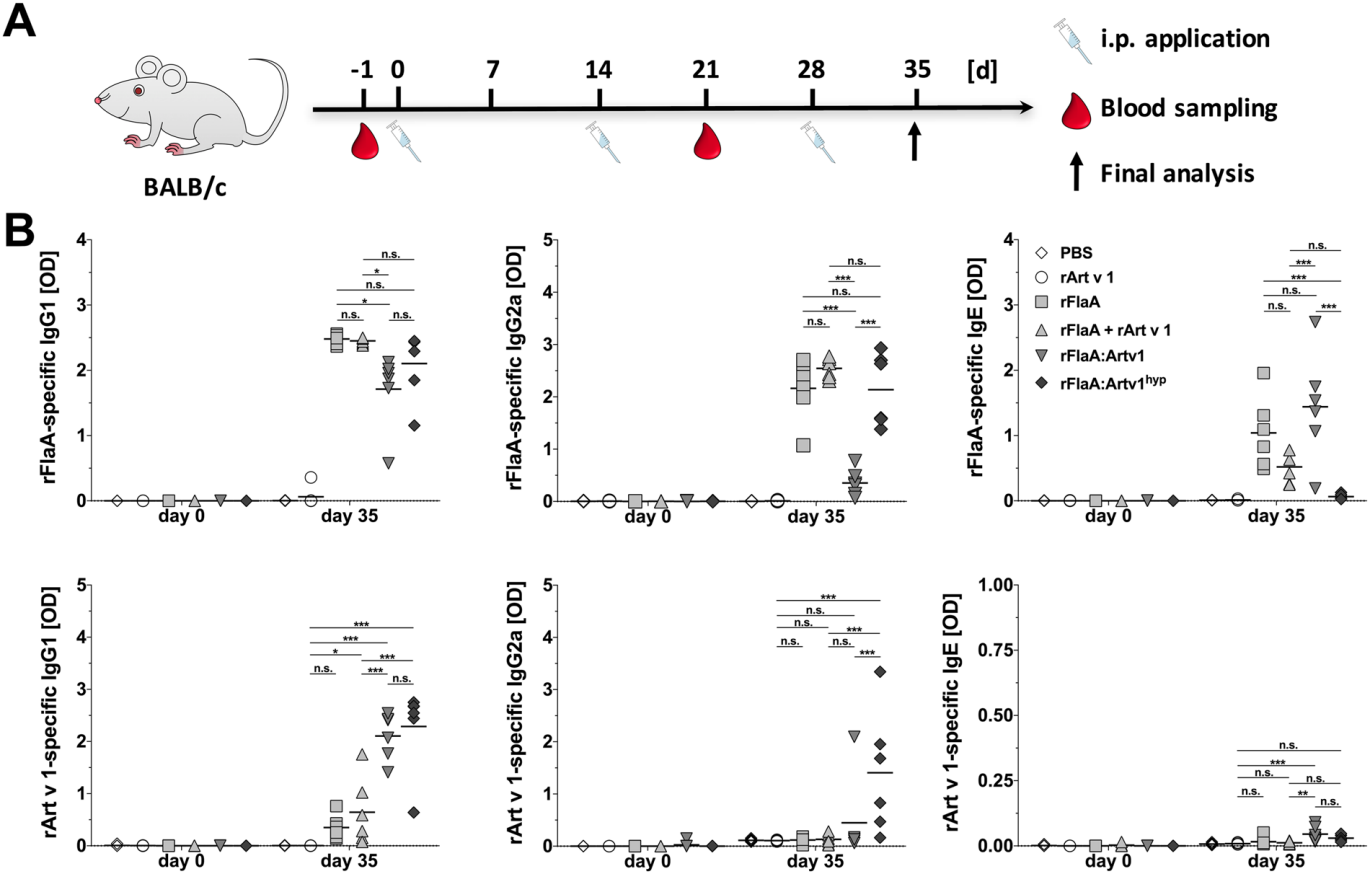
rFlaA:Artv1 and rFlaA:Artv1^hyp^ induce both rFlaA- and rArt v 1-specific antibody responses. BALB/c mice were injected three times every 14 days with the indicated proteins (all equimolar to 5 µg rArt v 1) or PBS (**A**). On days −1 and 35 serum samples were collected and analyzed for rFlaA- and Art v 1-specific IgG1-, IgG2a-, and IgE-levels by ELISA (**B**). Data are median values ± SD of two independent studies with 3 animals per group and study.

**Figure 7:**
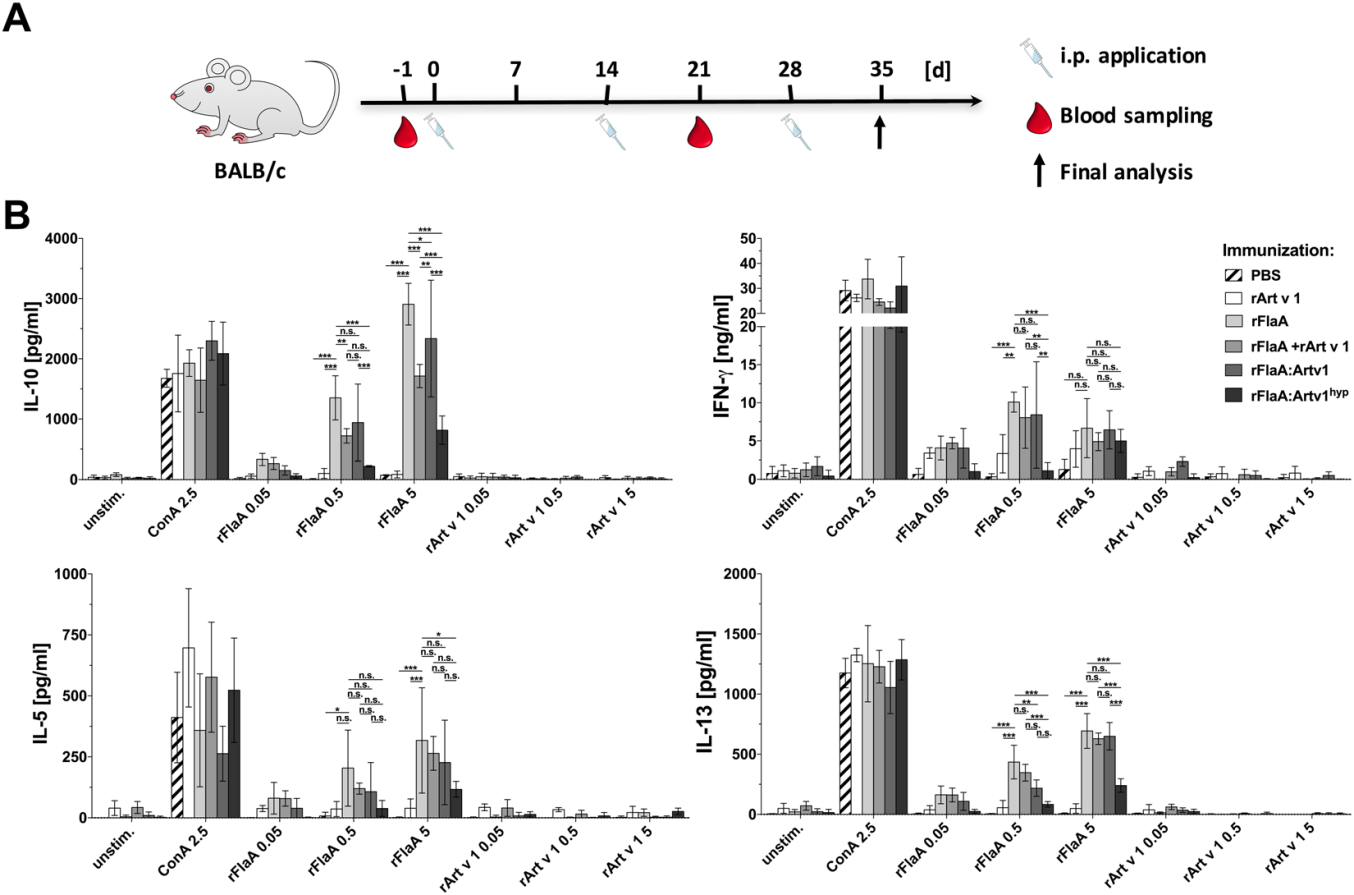
*In vivo* application of rFlaA:Artv1 and rFlaA:Artv1^hyp^ result in reduced TH2-cytokine secretion upon re-stimulation with rFlaA. BALB/c mice were injected three times every 14 days with the indicated proteins (all equimolar to 5 µg rArt v 1) or PBS (**A**). On day 35 splenocytes were isolated and re-stimulated with the indicated, equimolar amounts of rFlaA or rArt v 1. After 72 h, supernatants were collected and analyzed for levels of IL-5 and IL-13, IL-10, and IFN-γ (**B**). Data are mean values ± SD of two independent studies.

To investigate the immunogenicity of flagellin (in the absence of an additional adjuvant) we immunized BALB/c mice three times every 14 days by i.p. injection with equimolar amounts of rFlaA, rArt v 1, rFlaA + rArt v 1, rFlaA:Artv1, or rFlaA:Artv1^hyp^ (all equimolar to 5 µg rArt v 1) and determined both rFlaA- and rArt v 1-specific antibody titers (Fig. 6A) and cytokine secretion from splenocytes upon re-stimulation with rFlaA or rArt v 1 (Fig. 7).

While there were no differences in rFlaA-specific IgG1 levels between the groups that received rFlaA (either alone, in a mixture with rArt v 1, or as part of a fusion protein), application of rFlaA:Artv1 resulted in significantly lower levels of rFlaA-specific IgG2a production compared to all other groups (rFlaA, rFlaA + rArt v1, or rFlaA:Artv1^hyp^, Fig. 6B). Interestingly, rFlaA sensitization induced production of anti-FlaA IgE antibodies (Fig. 6B). Here, no significant differences in rFlaA-specific IgE levels were detected between animals that had received rFlaA, rFlaA + rArt v 1, or rFlaA:Artv1, while animals that had received rFlaA:Artv1^hyp^, did not produce rFlaA-specific IgE antibodies (Fig. 6B). Animals vaccinated with either rFlaA:Artv1 or rFlaA:Artv1^hyp^ developed significantly higher rArt v 1-specific IgG1 levels than the respective control groups (Fig. 6B). Interestingly, significantly increased levels of rArt v 1-specific IgG2a antibodies were only detected in animals that received rFlaA:Artv1^hyp^, but not rFlaA:Artv1 (Fig. 6B). None of the proteins was capable to induce rArt v 1-specific IgE antibodies (Fig. 6B). Finally, our data suggested that the application of both fusion proteins resulted in the predominant induction of TH1-biased immune responses.

In splenocyte cultures re-stimulation with rFlaA resulted in the production of both TH1- (IFN- γ) and TH2- (IL-5 and IL-13) cytokines as well as IL-10 production (Fig. 7B). Interestingly, re-stimulation with rFlaA of splenocytes from animals injected with rFlaA:Artv1^hyp^ resulted in the strongest reduction of IL-5 and IL-13 levels (Fig. 7B). In animals injected with rFlaA:Artv1^hyp^ re-stimulation with rFlaA also produced lower amounts of IL-10 and IFN-γ, while cytokine production from splenocytes of animals injected with rFlaA:Artv1 were not different to those observed from the splenocytes of animals that had received rFlaA or rFlaA + rArt v 1 (Fig. 7B). Here, no cytokine secretion was detected upon re-stimulation of splenocyte cultures with rArt v 1 (Fig. 7B).

### Both rFlaA:Artv1- and rFlaA:Artv1^hyp^–induced IL-10 secretion from mDCs is mediated by mTOR1 activation

**Figure 8:**
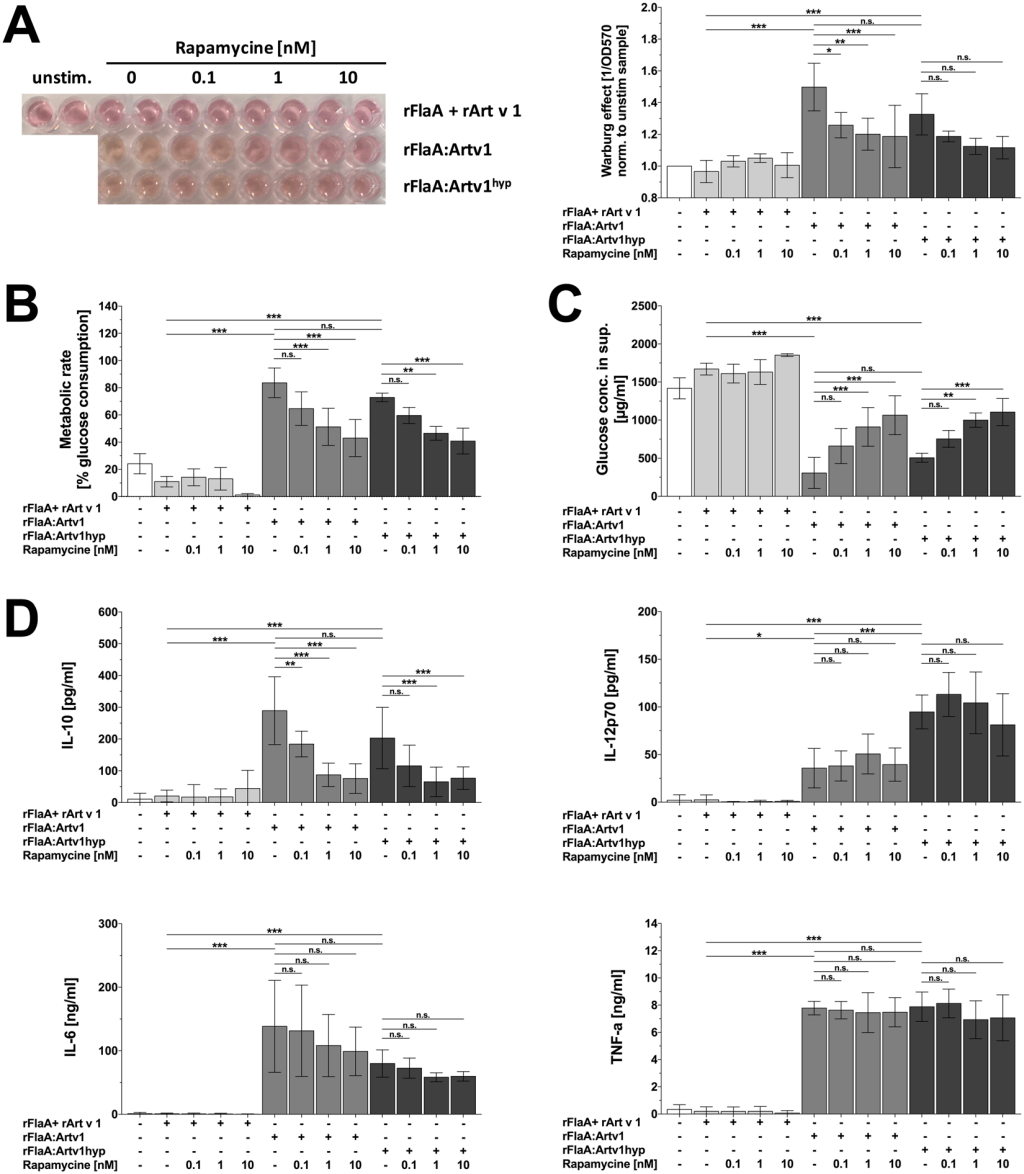
Both rFlaA:Artv1- and rFlaA:Artv1^hyp^–induced IL-10 secretion from mDCs is mediated by mTOR1-activation. BALB/c-bone marrow-derived mDCs were pretreated for 90 min with the indicated amounts of rapamycin and stimulated with either rFlaA + rArt v 1, rFlaA:Artv1, or rFlaA:Artv1^hyp^ for 72 h. Subsequently, the induced Warburg effect was quantified optically and photometrically (**A**), metabolic rate (**B**) and glucose consumption from culture medium (**C**) were determined and cytokine levels in culture supernatants were quantified by ELISA (**D**). Data are mean values ± SD of four independent experiments.

Next we addressed the mechanisms by which both fusion proteins modulate immune responses. Since previous studies^20,29^ suggested, that the immune modulating properties of flagellin fusion proteins strongly depend on mDC-derived IL-10 secretion we focused on the mechanism of IL-10 induction by both fusion proteins *in vitro* (Fig. 8).

We observed, that mDC cultures stimulated with rFlaA:Artv1 and rFlaA:Artv1^hyp^ for up to 72 h revealed a pronounced discoloration of the culture media, which was less pronounced in cells stimulated with the equimolar mixture of both proteins (Fig. 8A). This result suggested a strong metabolic activity of mDCs stimulated with both fusion proteins. This marked increase in overall cellular metabolism resulting from a predominant production of energy by a high rate of glycolysis and lactic acid fermentation is known as the Warburg effect^31^. When quantifying the Warburg effect in mDC cultures stimulated with rFlaA:Artv1 or rFlaA:Artv1^hyp^ compared to the non-fused mixture of rFlaA + rArt v 1 we observed, that both fusion proteins induced a significantly stronger Warburg effect than the equimolar mixture of both single proteins (Fig. 8A). In line with these results, also glucose consumption from the culture medium and therefore cell metabolic rates were strongly enhanced in mDCs stimulated with both fusion proteins but not the equimolar mixture of rFlaA + rArt v 1 (Fig. 8B,C). Taken together, these results suggested, that stimulation with both fusion proteins resulted in an activation of mDC metabolism. The mTOR1 complex is well described as a master regulator of both cellualr growth and immune responses. Previous results suggested, that flagellin fusion proteins may modulate mDC activation by inducing mTOR signalling (Schülke *et al*., 2017 JACI).

To verify that mTOR1 is involved in the increased metabolic activity we performed experiments in which we inhibited the mTOR1-complex by pre-treatment of the mDCs with rapamycin, a specific inhibitor of mTOR-activation (Fig. 8). In line with our hypothesis, mTOR-inhibition by rapamycin dose-dependently and significantly suppressed the induced Warburg effect (Fig. 8A), cell metabolic rates (Fig. 8B), glucose consumption from the culture medium (Fig. 8C), as well as IL-10 secretion induced by both fusion proteins (Fig. 8D), while pro-inflammatory IL-6 and TNF-α secretion were not affected by mTOR-inhibition (Fig. 8D). Of note, the Warburg effect and the IL-10 secretion induced by stimulation with rFlaA:Artv1^hyp^ was slightly (but not significantly) lower than observed for rFlaA:Artv1 (Fig. 8A,D). In line with the theory, that mTOR-induced IL-10 secretion suppresses IL-12p70 production, mDCs stimulated with rFlaA:Artv1^hyp^ produced significantly higher amounts of IL-12p70 compared to mDCs stimulated with rFlaA:Artv1 (Fig. 8D).

Taken together, these results show that rFlaA:Artv1 and rFlaA:Artv1^hyp^ modulate immune responses by an mTOR-dependent activation of mDC metabolism, resulting in immunosuppressive IL-10 secretion.

### rFlaA:Artv1 and rFlaA:Artv1^hyp^ induce dose-dependent IL-10 secretion from human DCs

**Figure 9:**
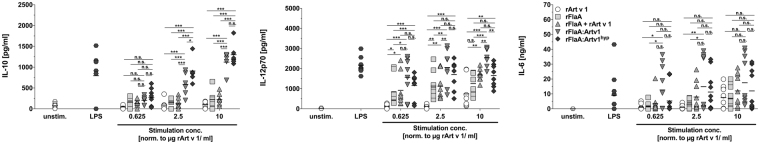
rFlaA:Artv1 and rFlaA:Artv1^hyp^ induce dose-dependent IL-10 secretion from human DCs. Human monocyte-derived DCs were differentiated from buffy coats of healthy donors and stimulated *in vitro* with LPS (1 ng/ml) or the indicated, equimolar amounts of Art v 1, rFlaA, rFlaA + Art v 1, rFlaA:Artv1, or rFlaA:Artv1^hyp^ for 24 h. Levels of IL-10, IL-6, and IL-12p70 in culture supernatants were quantified by ELISA. Data are mean values ± SD of eight different donors.

Finally, in an initial set of experiments, we determined the ability of the different constructs to activate human antigen presenting cells. For this, monocyte-derived DCs (moDC) were stimulated with both fusion proteins and the respective controls and checked for cytokine secretion by ELISA (Fig. 9). In accordance with the results obtained for mouse mDC we observed, that rFlaA:Artv1 and rFlaA:Artv1^hyp^ induced significantly higher levels of IL-10 secretion compared to rFlaA and Art v 1 alone or as a mixture (Fig. 9). Here, overall IL-10 levels induced by rFlaA:Artv1 and rFlaA:Artv1^hyp^ were comparable. In contrast, IL-6 secretion induced by both fusion proteins was not statistically different from levels observed for the respective controls. In human moDC IL-12p70 secretion was dose-dependently induced by stimulation with rFlaA, with levels being comparable in moDC stimulated with the mixture of rFlaA + Art v 1 (Fig. 9). Here, both rFlaA:Artv1 and rFlaA:Artv1^hyp^ induced only slightly higher levels of IL-12p70 secretion, which were only for rFlaA:Artv1-stimulated moDCs statistically different from levels observed after stimulating with the mixture of rFlaA and Art v 1 (Fig. 9).

In summary, both rFlaA:Artv1 and rFlaA:Artv1^hyp^ were able to induce a prominent secretion of the anti-inflammatory cytokine IL-10 from human dendritic cells, suggesting that these proteins might have the same immune modulating capacity on human cells that we observed in the mouse system.

## Discussion

### Characterization of FlaA fusion proteins and control proteins

Fusion proteins containing the TLR5-ligand flagellin are currently tested in clinical trials for the prevention of influenza infections^32,33^. Furthermore, such fusion proteins are considered as potential vaccine candidates for infectious diseases^24–27^, caries^28,34^, and allergies^20,29^.

In order to further optimize such vaccines candidates for the intervention of allergic diseases, we generated highly pure flagellin fusion proteins incorporating the major mugwort allergen Art v 1, both in its unmodified form as well as hypoallergenic derivative. To generate this hypoallergenic variant we substituted all cysteine residues involved in intramolecular disulfide bonds except Cys_26_ in order to maintain the previously identified immunodominant human TC epitope (KCIEWEKAQHGA at position 25 to 36)^10^. The mutation of cysteine residues disrupts intramolecular disulfide bonds and strongly reduces IgE reactivity while preserving T cell reactivity^30^. Consequently, both the hypoallergenic Art v 1 and rFlaA:Artv1^hyp^ did not show IgE reactivity upon incubation with sera from mugwort allergic patients.

Upon conjugation of the allergen to flagellin, flagellin dominated the CD spectra by its typical alpha-helical secondary structure elements. Here, the secondary structure of rArt v 1 and rArt v 1^hyp^ showed typical curve progressions for unfolded random coil structures. These findings are in accordance with the available literature showing that the hydroxyproline-rich part of Art v 1 is responsible for the generation of these random coil signals^3^. Analysis of protein aggregation showed, that rFlaA:Artv1 seemed to form several distinct high molecular aggregates, while rFlaA:Artv1^hyp^ displayed an uniform hydrodynamic radius corresponding to the expected size for a monomeric fusion protein. These differences in aggregation status between rFlaA:Artv1 and rFlaA:Artv1^hyp^ may be explained by differences in folding between both fusion proteins, likely caused by both the partial defolding of the fused allergen and the reduced capacity to form intra- and intermolecular disulfide bonds in the rFlaA:Artv1^hyp^ fusion protein. In case of rFlaA:Artv1 the defensin-like domain, which is stabilized by intramolecular disulfide bonds^3,30^, may allow for the structured assembly of flagellin in solution. Whereas the most likely unfolded random coil sequence of the hypoallergenic Art v 1 as part of the rFlaA:Artv1^hyp^ fusion protein may interfere with the self-assembly process of flagellin and the associated aggregation.

### Innate immune stimulatory capacity and mechanism of mDC activation

Despite differences in allergen sequence and aggregation status, the hypoallergenic rFlaA:Artv1^hyp^ retained its capacity to activate the target receptor TLR5 on TLR5-transfected reporter cells as well as the ability to activate both mouse (BALB/c- and C57Bl/6-derived) and human dendritic cells resulting in the secretion of both pro- and anti-inflammatory cytokines. Of note, both fusion proteins were able to induce high levels of the anti-inflammatory cytokine IL-10 from both murine and human dendritic cells. These results are in line with our own previous studies showing that a fusion protein consisting of rFlaA and the model allergen ovalbumin was able to induce a strong IL-10 secretion from murine dendritic cells^20,29^, preventing Ova-induced intestinal allergy^29^.

Mechanistically we were able to show the IL-10 secretion, but not the pro-inflammatory cytokine secretion, induced by both fusion proteins, to be inhibited by pre-treatment of the mDCs with rapamycin and therefore to be dependent on mTOR-activation. These results are in accordance with our recently published study where we describe a fusion protein of rFlaA and the major birch pollen allergen Bet v 1 (rFlaA:Betv1) to induce the activation of mammalian target of rapamycin (mTOR) which increased metabolic activity of the stimulated mDCs^35^. Here, as observed for rFlaA:Artv1 and rFlaA:Artv1^hyp^, rFlaA:Betv1-mediated IL-10 secretion, but not pro-inflammatory cytokine secretion, was inhibited by rapamycin in mDCs and therefore dependent on mTOR-activation^35^.

Flagellin itself has been described in the literature as effective mucosal adjuvant by enhancing the levels of B7-2 expression *in vivo*^36^, priming both NF-kappa B-mediated and pro-inflammatory-gene expression in intestinal epithelial cells via TLR5^37^, while also triggering the activation of the NLRC4 inflammasome and subsequent pro-inflammatory cytokine production^38,39^. Here, the promotion of adaptive immunity by flagellin can be effectively driven by either TLR5-mediated activation of NF-κB signaling or NLRC4-mediated inflammasome activation^40^. Besides epithelial cells^37^, CD11c(high)CD11b(high) lamina propria DCs in the small intestine were described as the main TLR5-expressing cells, secreting pro-inflammatory cytokines and inducing the differentiation of naive B cells^41,42^. More recently, flagellin-stimulation was also reported to result in rapid TLR5-dependent mTOR phosphorylation^43^. In this context we speculate, that the immunogenic properties of flagellin are retained in FlaA-containing fusion proteins.

### Suppression of TH2 immune responses

Interestingly, while both fusion proteins were able to suppress Art v 1-induced TH2 cytokine secretion from both *ex vivo* isolated TH2-biased CD4 T cells as well as in the *in vivo* vaccination model, only rFlaA:Artv1 containing the unmodified allergen sequence was able to efficiently induce IFN-γ secretion in mDC:TC co-cultures. In contrast, rFlaA:Artv1^hyp^ resulted in a prominent secretion of IL-10 not detected upon stimulation of co-cultures with rFlaA:Artv1. We hypothesize that the observed differences in T cell activation could be explained by either (I) differences in the allergen sequence between rFlaA:Artv1 and rFlaA:Artv1^hyp^, (II) differences in protein folding resulting from the disruption of intramolecular cysteine bonds introduced by our mutations, and/or (III) differences in protein uptake and processing due to the aforementioned differences in aggregation status between rFlaA:Artv1 and rFlaA:Artv1^hyp^.

While our own studies have shown that the (human) immunodominant T cell epitope of Art v 1 at position 25 to 36 is at least one of the epitopes relevant in mice (data not shown), we cannot exclude the disruption of other mouse T cell epitopes within the modified Art v 1 sequence of the rFlaA:Artv1^hyp^ fusion protein. However, we observed a robust induction of rArt v 1-specific IgG antibodies upon vaccination with rFlaA:Artv1^hyp^. Here, levels of rArt v 1-specific IgG antibodies were not different between rFlaA:Artv1 and rFlaA:Artv1^hyp^ vaccinated animals, showing that sufficient T cell epitopes were retained within the rFlaA:Artv1^hyp^ molecule to allow for efficient T cell-dependent B cell activation.

The present results provide further evidence that IL-10 secretion induced upon stimulation with flagellin fusion proteins is derived from dendritic cells and only to a very low extend from T cells^29^. Therefore, *in vivo* application of the fusion proteins was shown to result in suppression of TH2 responses directed against the fused allergen likely mediated by IL-10 production from antigen presenting cells early during vaccination (at the time of initial T cell activation by the fusion protein), but not for example from IL-10 producing regulatory T cells (Schülke, unpublished observation). These results are in line with our observation that IL-10 secretion induced upon *in vivo* application of a flagellin fusion proteins containing Bet v 1 is produced very rapidly with a maximum after 8 hours post injection and subsequent rapid decline in serum IL-10 levels^35^.

### Modulation of humoral immune responses

While, we did not observe Art v 1-specific antibody responses *in vivo* upon either vaccination with rArt v 1, the mixture of rFlaA + rArt v 1, or sensitization with rArt v 1 + Alum, analysis of antibody responses in animals vaccinated with rFlaA:Artv1 or rFlaA:Artv1^hyp^ showed that the activation of the innate immune system also resulted in the efficient induction of adaptive immune responses *in vivo*. In our experimental setting, at least 4 to 6 injections of either rArt v 1 + Alum or rArt v 1 + Adjuphos are required, in order to induce rArt v 1-specific antibody production (Kuttich, unpublished observation). Therefore, two i.p. injections of rArt v 1 + Alum are likely not sufficient to induce a substantial rArt v 1-specific antibody production but sufficient to induce rArt v 1-specific TH2 responses. In contrast, the induction of rArt v 1-specific IgG1 and IgG2a antibodies was only detected in sera of animals vaccinated with rFlaA:Artv1 and rFlaA:Artv1^hyp^, but not the respective controls, suggesting the induction of allergen-specific TH1-biased immune responses *in vivo*. Additionally, the induction of rFlaA-specific antibodies was retained for both fusion proteins. In the context of allergy treatment, the observed induction of allergen-specific IgG2a antibodies is of particular interest, since these antibodies likely act as blocking antibodies preventing allergic reactions^44^.

In summary, we showed that flagellin:allergen fusion proteins incorporating the major mugwort allergen Art v 1, both in its wild type form and as a hypoallergenic variant, are potent vaccine candidates efficiently suppressing allergen-specific TH2 cytokine secretion both *in vitro* and *in vivo* while inducing an mTOR-dependent IL-10 secretion from mDCs. Interestingly, incorporation of the hypoallergenic Art v 1 variant into the flagellin fusion protein, did not interfere with its innate immune stimulatory potential, retaining its capacity to induce secretion of high levels of mTOR-dependent, anti-inflammatory IL-10 from murine and human dendritic cells. Therefore, flagellin:allergen fusion proteins incorporating hypoallergenic allergen variants are promising tools to further improve efficacy and safety of these innovative vaccine candidates for the treatment of allergies.

## Material and Methods

### Generation of Proteins

For recombinant generation of the major mugwort (*Artemisia vulgaris*) pollen allergen Art v 1.0101 sequence information was obtained from GenBank (Acc. No: AF493943). Cloning of the fusion proteins was performed using synthetic genes (GeneArt, Regensburg, Germany) encoding for the rFlaA sequence (Acc. No: ×65624) at the N-terminal part and Art v 1/Art v 1^hyp^ sequences at the C-terminal part, respectively. For the generation of the hypoallergenic fusion protein rFlaA:Artv1^hyp^ and its respective control Art v 1^hyp^, 7 out of 8 total cysteines were exchanged to serines in order to destroy the described disulfide bonds contributing to conformational IgE-epitopes of the allergen^30^. The cysteine residue present in the immunodominant human T cell epitope (at AA-positions 25–36^10^) remained unmodified.

The following oligonucleotide sequences were used for amplification of the indicated sequences: rFlaA:Artv1 forward sequence (fw): CGCGCGGCAGCCATATGAAAGTGAACACCAACATC, rFlaA:Artv1 reverse sequence (rev): CAGCCGGATCCTCGAGTTAATGGGTGCTCGGTGGC; rFlaA:Artv1^hyp^ fw: CGCGCGGCAGCCATATGAAAGTGAACACCAACATC,rFlaA:Artv1^hyp^ rev: CAGCCGGATCCTCGAGTTAATGGGTGCTCGGTGGC; rArt v 1 fw: CGCGCGGCAGCCATATGGCAGGTAGCAAACTGTGTG rArt v 1 rev: CAGCCGGATCCTCGAGTTAATGGGTGCTCGGTGGC, rArt v 1^hyp^ fw: CGCGCGGCAGCCATATGGCAGGTAGCAAACTGTCTG rArt v 1^hyp^ rev: CAGCCGGATCCTCGAGTTAATGGGTGCTCGGTGGC. cDNA sequences were confirmed by bidirectional Sanger sequencing (MWG Eurofins, Ebersberg, Germany). All inserts were cloned into pET15b by InFusion® cloning (Clontech, Heidelberg, Germany) and expressed in *E*. *coli* BL21(DE3) (rFlaA, rFlaA:Artv1, rFlaA:Artv1^hyp^) or *E*. *coli* Rosetta-gami^TM^ B(DE3)pLysS (rArt v 1 and rArt v 1^hyp^) overnight at 37 °C (rFlaA:Artv1 and rFlaA:Artv1^hyp^) or 20 °C (Art v 1 and Art v 1^hyp^) using 1 mM IPTG (Fermentas, St. Leon-Roth, Germany, rFlaA:Artv1 and rFlaA:Artv1^hyp^) or 0.4 mM IPTG (Art v 1 and Art v 1^hyp^) for induction. Cell lysis was performed according to Schülke *et al*.^45^. Proteins were purified from bacterial extracts by either a combination of immobilized metal ion affinity chromatography and subsequent size exclusion chromatography (rFlaA:Artv1 and rFlaA:Artv1^hyp^) or filtration via a PES-membrane using 50 ml Falcon filtration system (50 kDa cut-off, Sartorius, Göttingen, Germany) followed by cation exchange chromatography (Art v 1 and Art v 1^hyp^) on an ÄKTA FPLC system (GE Healthcare, Munich, Germany). All proteins were dialyzed against PBS (pH = 7.4) for further experiments. Recombinant flagellin A from *Listeria monocytogenes* (rFlaA, GenBank Acc. No: × 65624) was generated according to^45^. For all proteins endotoxin content was depleted after final protein purification using size exclusion chromatography in combination with either the EndoTrap®blue system (Hyglos, Bernried, Germany) (rFlaA, rArt v 1, rFlaA:Artv1, rFlaA:Artv1^hyp^) or as described by Bordier *et al*.^46^ (rArt v 1, rArt v 1^hyp^). Protein concentrations were determined using the BCA Protein Assay Kit (Thermo Scientific, Dreieich, Germany). Total yields for final protein preparations were 6.5 mg rArt v 1, 3 mg rArt v 1^hyp^, 26 mg rFlaA, 13 mg rFlaA:Artv1, and 12 mg rFlaA:Artv1^hyp^. All proteins displayed a purity greater than 98%.

### SDS-PAGE

SDS-PAGE was performed according to the method described by Laemmli^47^ (cross linker C = 5%, total bis/acrylamid 15%) under reducing conditions.

### Confirmation of sequence identity by LC-MS^E^

Recombinant rFlaA:Artv1 fusion protein was separated by SDS-PAGE and stained with Coomassie Brilliant Blue. Visible protein bands were excised, in-gel digested using trypsin and analyzed by mass spectrometry (nano-UPL nanoESI-MS^E^ as published previously^48^. Differing to this study, MS^E^ data were searched against a UniProt database consisting of reviewed entries of eucaryotic organisms (taxon identifier 2759) and the amino acid sequence of rFlaA:Artv1 fusion proteins.

### LAL-assay

Endotoxin concentration was determined via chromogenic *Limulus* amebocyte lysate (LAL) assay according to the manufacturers’ recommendations (Endosafe® PTS LAL system, Charles River, Sulzfeld, Germany). The different proteins displayed the following endotoxin contents: <1,14 pg/µg protein (rFlaA), <3.7 pg/µg protein (rArt v 1), <1.5 pg/µg protein (rArt v 1^hyp^), <3 pg/µg protein (rFlaA:Artv1) and <2 pg/µg protein (rArt v 1^hyp^), respectively.

### Patients and sera

Sera of mugwort pollen-allergic individuals were selected based on typical case history, i.e. periodic rhinitis/conjunctivitis during late summer and allergen-specific IgE (CAP-FEIA ≥3) to mugwort pollen (w6; Phadia, Uppsala, Sweden). The study and all used experimental protocols were approved by the ethics committee of the Medical University of Vienna (EK No. 497/2005), all methods were carried out in accordance with the relevant guidelines and regulations. Informed consent was obtained from all subjects.

### Western Blot

For IgE immunoblot analysis, proteins were separated by SDS-PAGE and blotted onto nitrocellulose Protran® membranes (Whatman, Dassel, Germany) according to Towbin *et al*.^49^. Sera from mugwort allergic patients and non-allergic controls were diluted 1:6 or 1:10 in 100 mM Tris-HCl (pH = 7.4), 100 mM NaCl, 2.5 mM MgCl_2_, 0.05% (v/v) Tween20, 0.1% (w/v) BSA. Bound IgE was detected using a mouse-anti-human IgE-alkaline phospathase (AP) conjugated antibody (BD Pharmingen, Heidelberg, Germany) and visualized with AP Conjugate Substrate Kit (Bio-Rad, Munich, Germany).

### Circular dichroism spectroscopy

Purified proteins were adjusted to a concentration of 200 µg/ml and dialyzed against 10 mM phosphate buffer. Circular dichroism (CD) spectra were recorded using a JASCO J-810 spectropolarimeter (Jasco, Gross-Umstadt, Germany) with constant N_2_ flushing at 20 °C. Measurements were performed in a quartz glass cuvette (1 mm, Hellma, Müllheim, Germany) with a step width of 1 nm and a band width of 1 nm. The spectral range was 180–260 nm at 50 nm/min. Ten scans were accumulated, spectra obtained with buffer were subtracted. The results were expressed as mean residue molar ellipticity [H]_MRD_ and served as indication for formation of secondary structure elements.

### Dynamic light scattering analysis

Dynamic light scattering analysis was performed using a Zetasizer Nano ZS (Malvern, Herrenberg, Germany) at room temperature. Three individual measurements per sample were performed and the mean frequencies (calculated as relative % in class) of hydrodynamic radii (R_H_) in nm were plotted.

### TLR5-activation assay

HEK 293 cells stably transfected with the murine TLR5 (InvivoGen, Toulouse, France) were cultured in DMEM (Biochrome, Berlin, Germany) containing 10% FCS (Biochrom, Berlin, Germany), L-glutamine (0.15 mg/ml), penicillin (100 U/ml), streptomycin (100 µg/ml), and blasticidin (10 µg/ml) (InvivoGen). Wild-type HEK 293 cells were cultured without blasticidin as indicated above. For TLR5-activation assays, 4 × 10^4^ cells per well were seeded in 48-well plates and cultured in DMEM containing 2% FCS, L-glutamine, penicillin, and streptomycin overnight. Cells were stimulated for 22 h with equimolar amounts rFlaA, rFlaA:Artv1, or rFlaA:Artv1^hyp^. Subsequently, human IL-8 secreted in the supernatant was quantified by ELISA using the BD OptEIA human IL-8 ELISA Set (BD Biosciences, Heidelberg, Germany).

### Mice

BALB/c mice (Charles River Laboratories, Sulzfeld, Germany,) were kept at the animal facility of the Paul-Ehrlich-Institut under specific pathogen-free conditions. All animal experiments were performed in compliance with the German animal protection law (granting authority: RP Darmstadt, Germany, Approval number: F107/131).

### *In vitro* generation of mouse bone marrow-derived dendritic cells

Mouse myeloid dendritic cells (mDCs) were generated as described previously^45^. Briefly, bone marrow cells (BMCs) were isolated from femur and tibia of BALB/c mice and differentiated into mDCs using GM-CSF (R&D Systems, Minneapolis, USA). On day eight, mDCs were harvested for experiments.

### Dendritic cell stimulation and flow cytometry

BALB/c or C57Bl/6 mDCs were seeded at 3.2 × 10^5^ cells/ml in 24-well plates (Thermo Scientific, Dreieich, Germany) and stimulated with the indicated equimolar concentrations of rFlaA, rArt v 1, rFlaA + rArt v 1, rFlaA:Artv1, or rFlaA:Artv1^hyp^ for 24 h. 10 µg/ml LPS (#L5886, Sigma Aldrich, Taufkirchen, Germany) served as positive control. Supernatants were analyzed for cytokine secretion by ELISA. The activation of mDCs was assessed by FACS using anti-mouse FITC-conjugated CD40 and phycoerythrin (PE)-conjugated CD69 mAbs (eBioscience, Frankfurt, Germany). Additionally, cells were stained with anti-mouse pacific blue-conjugated CD11b (Invitrogen, Thermo Fisher Scientific), allophycocyanin (APC)-conjugated CD11c (BD Bioscience, Heidelberg, Germany), and PE-Cy5-conjugated B220 (BD Bioscience) with their respective isotype controls. FITC or PE intensity of CD11b^+^CD11c^+^B220^−^ (mDC) cells was quantified by FACS using a LSR II flow cytometer (BD Bioscience, Heidelberg, Germany). For analysis of cell viability mDC were treated as indicated and stained for dead cells using the fixable viability dye eFlour450 (eBiosciences, Frankfurt, Germany). Data were analyzed using FlowJo 10 (Treestar Inc., Ashland, OR, USA).

### Cytokine ELISAs

Human IL-8, murine IL-2, IL-6, IL-10, IL-12p70, IL-13 and IFN-γ in culture supernatants were quantified using the BD OptEIA™ ELISA Sets (BD Biosciences, Heidelberg, Germany). Human IL-6, IL-10 and IL-12p70 levels in supernatants of stimulated monocyte-derived DCs were determined using the PeproTech human ELISA Kits (Pepro Tech, Hamburg, Germany) according to the manufacturers’ recommendations.

### Preparation of mDC and CD4^+^ T cell co-cultures

Splenic CD4^+^ T cells were isolated from female BALB/c mice (age 10 weeks) immunized with rArt v 1 plus aluminium hydroxide and magnesium hydroxide (Imject, Thermo Scientific, Dreieich, Germany) (2 times 10 µg Art v 1 + 2 mg Alum in 200 µl PBS i.p. two weeks apart) using the CD4^+^ T Cell Isolation Kit (Miltenyi Biotec, Bergisch Gladbach, Germany). BALB/c mDCs were either cultured alone (0.8 × 10^6^ cells/mL) or with CD4^+^ T cells (3.2 × 10^6^ cells/mL, >95% purity) and stimulated with the different proteins for 24 or 72 h. Subsequently, concentrations of IL-2, IL-10, IL-13 and IFN-γ in the supernatants were measured by BD OptEIA™ ELISA (BD Biosciences).

### Prophylactic vaccination experiments

For prophylactic vaccination experiments female BALB/c mice (n = 6 mice/group) were treated twice intraperitoneal (i.p.) with the indicated proteins (rFlaA, rArt v 1, rFlaA + rArt v 1, rFlaA:Artv1, rFlaA:Artv1^hyp^ (all equimolar to 10 µg rArt v 1), or PBS, followed by two i.p.-sensitizations with Art v 1 + Alum (10 µg rArt v 1 plus 2 mg Alum in 200 µl PBS i.p.) in a 2 week interval. At days 0 and 41 serum samples were collected and analyzed for Art v 1-specific IgG1-, IgG2a-, and IgE-levels. On day 42, CD4^+^ TCs were isolated using the CD4^+^ T Cell Isolation Kit (Miltenyi Biotec, Bergisch Gladbach, Germany) and co-cultured with BALB/c bone-marrow-derived mDC according to the section “Preparation of mDC and CD4^+^ T cell co-cultures” and re-stimulated *in vitro* with rArt v 1. After 24 and 72 h, supernatants were collected and analyzed for levels of IL-2, IL-10, IL-13, and IFN-γ (IL-4 and IL-5 data not shown) by ELISA.

### Determination of Art v 1-specific antibody titers

For determination of Art v 1-specific IgG1, IgG2a, and IgE antibody titers in mouse sera, 96-well Nunc™Maxisorp™ ELISA plates (Thermo Scientific, Dreieich Germany) were coated with 100 µl of a 10 µg/ml Art v 1 solution in coating buffer (50 mM NaCO_3_, pH = 9.6) overnight at 4 °C. Serum samples were diluted by serial dilution (for IgE: 1 × 1:10, then 6 × 1:2 in 50 µl/well, for IgG1 and IgG2a: 1 × 1:100, then 6 × 1:2 in 100 µl/well) and incubated at 4 °C overnight (IgE) or for 2 h at room temperature (IgG1, IgG2a). Levels of Art v 1-specific antibodies were measured using 50 µl/well secondary detection antibody diluted in PBS 10% FCS (IgE: rat anti-mouse IgE biotin-conjugated, 1:1000, #553419, BD Pharmingen Biosciences, Heidelberg, Germany; IgG1: goat anti-mouse IgG_1_ gamma 1 HRP-conjugated, 1:8000, #A10551; IgG2a: rabbit anti-mouse IgG_2a_ HRP-conjugated, 1:8000, #610220, both Invitrogen, Darmstadt, Germany; incubation times: 1.5 h for IgG1 and IgG2a, 1 h for IgE). For IgE detection, samples were further incubated with a streptavidin-HRP antibody (50 µl diluted 1:2000 in PBS 10% FCS, BD Biosciences) for 30 minutes at room temperature. Detection was performed with 100 µl/well TMB substrate solution for up to 30 minutes. The reaction was stopped by addition of 50 µl/well 25% H_2_SO_4_ and analyzed using either a SpectraMAX340PC or a SpectraMAX384Plus (Molecular Devices, California, USA) reading the absorbance at 450 nm. Data were analyzed using GraphPad Prism 5–7 for Mac or Windows.

### Analysis of rFlaA- and rArt v 1-specific immune responses upon *in vivo* application

To gain further insight in the immune responses induced by flagellin and rArt v 1 BALB/c were immunized mice three times every 14 days by i.p. injection with equimolar amounts of rFlaA, rArt v 1, rFlaA + rArt v 1, rFlaA:Artv1, or rFlaA:Artv1^hyp^ (all equimolar to 5 µg rArt v 1 per mouse in 200 µl PBS, without adjuvant). Two weeks after the third sensitization mice were sacrificed and 3 × 10^6^ splenocytes were re-stimulated with Concanavalin A (Sigma-Aldrich), or either rFlaA or rArt v 1 (equimolar to 0.05, 0.5, or 5 µg/ml rArt v 1) for 72 h and analyzed for cytokine secretion by ELISA using BD OptEIA™ ELISA Sets (BD Biosciences). rFlaA- and rArt v 1-specific IgG1, IgG2a and IgE levels were determined by ELISA.

### Analysis of cell metabolic state

The Warburg effect in stimulated mDC cultures was either documented by photographing the culture plates or determined photometrically 72 h post stimulation by quantifying the OD at 570 nm and calculating the Warburg effect as 1/OD_570_ normalized to the unstimulated control. For inhibitor experiments mDCs were preincubated with the indicated amounts of rapamycin, (Invivogen, Toulouse, France) for 90 minutes and subsequently stimulated with equimolar amounts of rFlaA + rArt v 1, rFlaA:Artv1, or rFlaA:Artv1^hyp^ for 72 h. Glucose concentrations in culture supernatants were determined 72 h post-stimulation using the Glucose (GO) Assay Kit (Sigma-Aldrich). The metabolic rate was derived mathematically from the measured glucose concentrations by calculating the glucose consumption in % of medium without mDCs (glucose conc. in RPMI1640 = 2 mg/ml).

### Generation of human DCs

Human monocyte-derived DCs (moDCs) were differentiated from buffy coats of healthy donors according to Sender *et al*.^50^. Briefly, human peripheral blood mononuclear cells (PBMCs) were isolated by Ficoll (Histopaque 1077, Sigma Aldrich, Steinheim, Germany) density gradient centrifugation from buffy coats (commercially obtained from: Blutspendedienst, Frankfurt am Main, Germany). Purification of monocytes was performed by positive selection using CD14 MicroBeads (Miltenyi Biotech). For generation of moDCs, 1 × 10^6^ cells/ml were seeded in 24-well plates (Thermo Scientific) in complete medium (RPMI 1640 (Sigma-Aldrich), 10% FCS, 10 mM HEPES, 2 mM L-Glutamine (Biochrome), 100 U Penicillin/Streptomycin (Biochrome), 25 mM beta-mercapto ethanol) supplemented with 5 ng/ml GM-CSF (Leukine, Sanofi-Aventis, Frankfurt, Germany) and 10 ng/ml IL-4 (CellGenix, Freiburg, Germany). Cells were differentiated at 37 °C and 5% CO_2_ for five days. Human moDCs were harvested on day 5 and stimulated with the indicated, equimolar amounts of the different proteins for 24 h. 1 ng/ml LPS (Sigma Aldrich) served as positive control. Supernatants were analyzed for cytokine secretion by ELISA.

### Statistical analysis

Statistical analysis was performed with GraphPad Prism 5–7 for Mac or Windows using 2-way ANOVA tests with confidence intervals adjusted for multiple comparisons according to Bonferroni. For statistically significant results the following convention was used: *p-value < 0.05, **p-value < 0.01, ***p-value < 0.001.

## Electronic supplementary material

Supplementary information
