# Author Correction: Conjugation of wildtype and hypoallergenic mugwort allergen Art v 1 to flagellin induces IL-10-DC and suppresses allergen-specific TH2-responses *in vivo*

**DOI:** 10.1038/s41598-018-20635-3

**Published:** 2018-02-06

**Authors:** Stefan Schülke, Kirsten Kuttich, Sonja Wolfheimer, Nadine Duschek, Andrea Wangorsch, Andreas Reuter, Peter Briza, Isabel Pablos, Gabriele Gadermaier, Fatima Ferreira, Stefan Vieths, Masako Toda, Stephan Scheurer

**Affiliations:** 1 Section Molecular Allergology, Paul-Ehrlich-Institut, Langen, Hessen Germany; 2 Division of Allergology, Paul-Ehrlich-Institut, Langen, Hessen Germany; 3 Department of Molecular Biology, Division of Allergy and Immunology, University of Salzburg, Salzburg, Austria

Correction to: *Scientific Reports* 10.1038/s41598-017-11972-w, published online 18 September 2017

The original version of this Article contains errors in the spelling of the authors Stefan Schülke, Kirsten Kuttich, Sonja Wolfheimer, Nadine Duschek, Andrea Wangorsch, Andreas Reuter, Peter Briza, Isabel Pablos, Gabriele Gadermaier, Fatima Ferreira, Stefan Vieths, Masako Toda & Stephan Scheurer, which were incorrectly given as Schülke Stefan, Kuttich Kirsten, Wolfheimer Sonja, Duschek Nadine, Wangorsch Andrea, Reuter Andreas, Briza Peter, Pablos Isabel, Gadermaier Gabriele, Ferreira Fatima, Vieths Stefan, Toda Masako & Scheurer Stephan.

These errors have now been corrected in the PDF and HTML versions of the Article, and in the accompanying Supplementary Material.

