# Supplementary material for: Conjugation of wildtype and hypoallergenic mugwort allergen Art v 1 to flagellin induces IL-10-DC and suppresses allergen-specific TH2-responses in vivo
Source: Sci Rep. 2017 Sep 18;7:11782. doi: 10.1038/s41598-017-11972-w (PMC5603567; doi:10.1038/s41598-017-11972-w)
Supplement: Supplementary file 1 — Supplementary information [file 41598_2017_11972_MOESM1_ESM.doc]

**Conjugation of wildtype and hypoallergenic mugwort allergen Art v 1 to flagellin induces IL-10-DC and suppresses allergen-specific TH2-responses *in vivo***

Stefan Schülke^1*+^, Kirsten Kuttich^1+^, Sonja Wolfheimer^1^, Nadine Duschek^1^, Andrea Wangorsch^1^, Andreas Reuter^2^, Peter Briza^3^, Isabel Pablos^3^, Gabriele Gadermaier^3^, Fatima Ferreira^3^, Stefan Vieths^1^, Masako Toda^1^, Stephan Scheurer^1^

^1^Section Molecular Allergology and ^2^Division of Allergology, Paul-Ehrlich-Institut, Langen, Hessen, Germany; ^3^Department of Molecular Biology, Division of Allergy and Immunology, University of Salzburg, Salzburg, Austria

^+^Authors equally contributed to the study

**Short title:** flagellin:Artv1 fusion proteins

***Corresponding author:**

Stefan Schülke, PhD

Paul-Ehrlich-Institut, Vice President´s Research Group 1: Molecular Allergology

Paul-Ehrlich-Str. 51-59, 63225 Langen, Germany

phone: +49 6103 77 5209

fax: +49 6103 771258

email: [Stefan.Schuelke@pei.de](mailto:Stefan.Schuelke@pei.de)

**Supplementary Figures:**

**Supplementary Figure S1: rFlaA:Artv1 and rFlaA:Artv1^hyp^ potently activate C57Bl/6 mDC leading to pro- and anti-inflammatory cytokine secretion.** C57Bl/6 bone marrow-derived mDCs were stimulated *in vitro* with equimolar amounts of rFlaA+rArt v 1, rFlaA+rArt v 1^hyp^, rFlaA:Artv1, or rFlaA:Artv1^hyp^ for 24 h and cytokine levels in culture supernatants were quantified by ELISA. Data are mean values ±SD of two independent experiments.

**
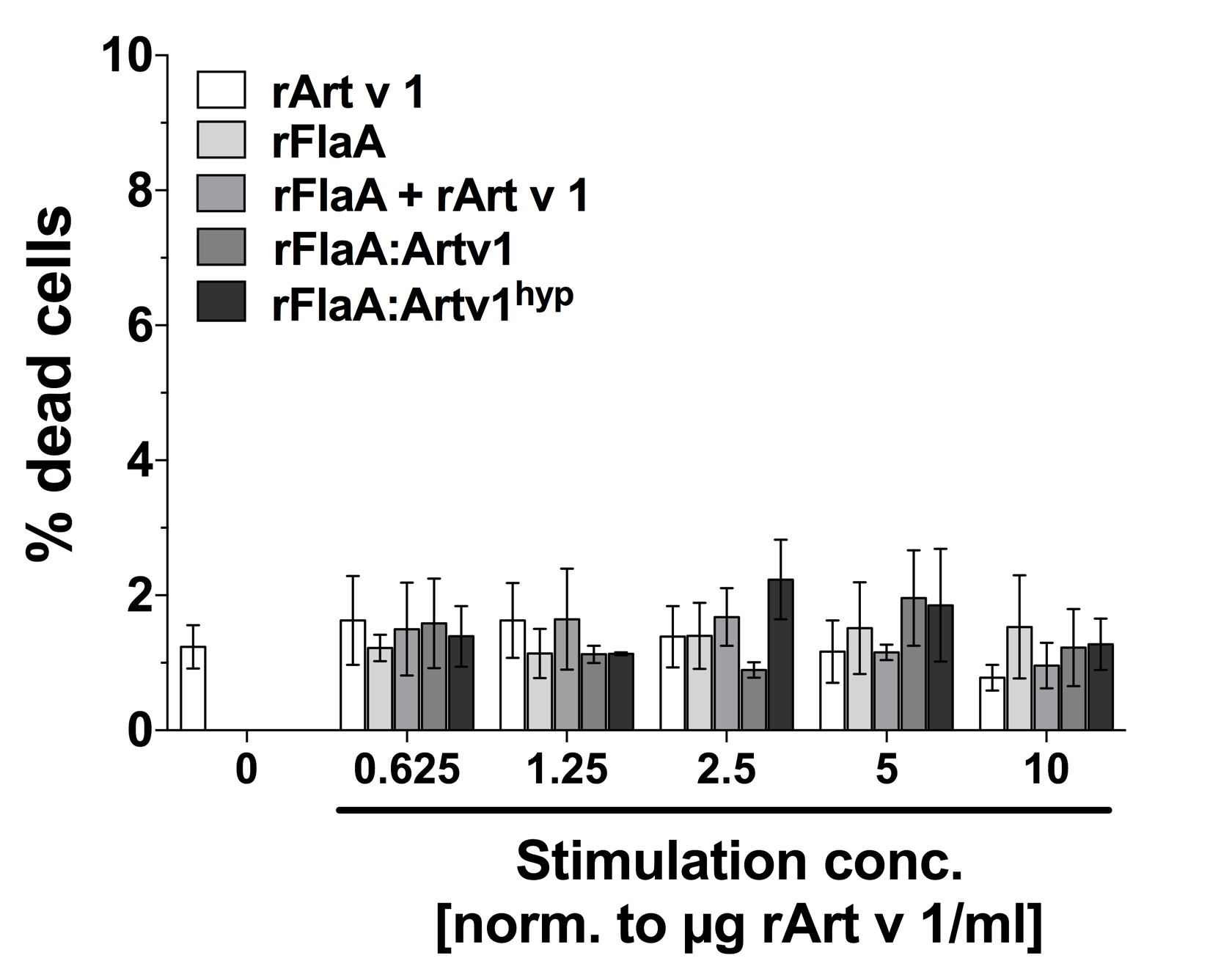
**

**Supplementary Figure S2: Stimulation with fusion proteins has no toxic effect on mDCs.** BALB/c-bone marrow-derived mDCs were stimulated *in vitro* with equimolar amounts of rArt v 1, rFlaA, rFlaA+rArt v 1, rFlaA:Artv1, or rFlaA:Artv1^hyp^ for 24 h and the percentage of dead cells was determined using fixable viability dye by flow cytometry. Data are mean values ±SD of three independent experiments.

**Supplementary Figure S3: Both** **fusion proteins suppress TH2-cytokine production in a co-culture of mDC and Art v 1-specific, TH2-biased T cells.** BALB/c bone marrow-derived mDCs were co-cultured with *ex vivo*-isolated CD4^+^ T cells from Art v 1-immunized BALB/c mice and stimulated with the indicated proteins in the absence of additional rArt v 1-re-stimlation to induce Ag-specific recall responses. Levels of IL-2 in culture supernatants were determined 24 h post-stimulation, IFN-γ, IL-13, and IL-10 levels 72 h post-stimulation by ELISA. Data are mean values ±SD of three independent experiments.

**Supplementary Figure S~~4~~: Art v 1-induced IL-5 secretion is suppressed after vaccination with rFlaA:Artv1 and rFlaA:Artv1^hyp^.** BALB/c mice were treated two times (i.p.) with the indicated proteins (all equimolar to 10 µg rArt v 1) or PBS, followed by two i.p.-sensitizations with Art v 1+Alum in a 2-week interval. CD4^+^ TCs were isolated on day 42 and co-cultured with BALB/c bone-marrow-derived mDC and re-stimulated *in vitro* with rArt v 1. After 72 h, supernatants were collected and analyzed for levels of IL-5. Spleens of three animals per group were pooled for the isolation of CD4^+^ T cells. Data are mean results of two independent studies ±SD.

**
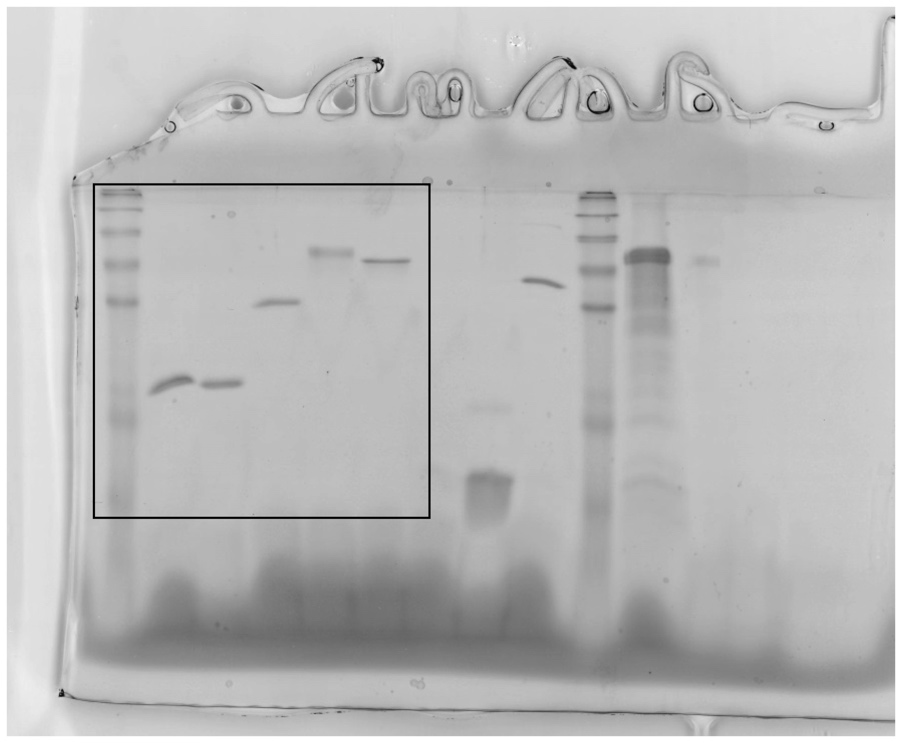
**

**Supplementary figure S5: Raw SDS-PAGE data used to generate Fig. 1A.** Coomassie staining of final protein preparations. The box indicates the part of the SDS-PAGE that was used to generate Fig. 1A.
